# Brazilian cardio-oncology: the 10-year experience of the Instituto do Cancer do Estado de Sao Paulo

**DOI:** 10.1186/s12872-020-01471-8

**Published:** 2020-04-28

**Authors:** Isabela B. S. da S. Costa, Cristina S. Bittar, Silvia M. R. Fonseca, Carolina M. P. D. e Silva, Marilia H. H. dos Santos Rehder, Stéphanie I. Rizk, Cecilia B. B. V. Cruz, Clara S. Figueiredo, Fernanda T. de A. Andrade, Ludmila de A. Barberino, Fernanda A. de S. Costa, Letticya P. Machado, Thalita B. González, Marcel P. C. Almeida, Julia T. Fukushima, Roberto Kalil Filho, Ludhmila Abrahao Hajjar

**Affiliations:** 1 Instituto do Câncer do Estado de São Paulo, Faculdade de Medicina da Universidade de São Paulo, Dr Arnaldo avenue, 251, São Paulo, 01246-000 Brazil; 2 Instituto do Coração, Faculdade de Medicina da Universidade de São Paulo, São Paulo, Brazil

**Keywords:** Cardio-oncology, Cancer, Cardiotoxicity

## Abstract

**Background:**

In recent years, the field of cardio-oncology has grown worldwide, bringing benefits to cancer patients in terms of survival and quality of life. This study reports the experience of a pioneer cardio-oncology programme at University Cancer Hospital in Brazil over a period of 10 years, describing the clinical profile of patients and the clinical outcomes.

**Methods:**

A retrospective study was conducted on a cohort of patients treated at the cardio-oncology programme from April 2009 to February 2019. We analysed the characteristics of patients and outcomes, including mortality, according to the type of clinical indication for outpatient care (general cardiology, perioperative evaluation and follow-up and treatment cardiotoxicity).

**Results:**

From a total of 26,435 medical consultations, we obtained the data of 4535 individuals among the medical care outpatients. When we analysed the clinical characteristics of patients considering the clinical indication - general cardiology, perioperative evaluation and cardiotoxicity outpatient clinics, differences were observed with respect to age (59 [48–66], 66 [58–74] and 69 [62–76], *p* < 0.001), diabetes (67 [15%], 635 [22.6%] and 379 [29.8%]; *p* < 0.001), hypertension (196 [43.8%], 1649 [58.7%] and 890 [70.1%], *p* < 0.001) and dyslipidaemia (87 [19.7%), 735 [26.2%] and 459 [36.2%], *p* < 0.001). A similar overall mortality rate was observed in the groups (47.5% vs. 45.7% vs. 44.9% [*p* = 0.650]).

**Conclusion:**

The number of oncologic patients in the Cardio-Oncology Programme has grown in the last decade. A well-structured cardio-oncology programme is the key to achieving the true essence of this area, namely, ongoing care for cancer patients throughout the disease treatment process, optimizing their cardiovascular status to ensure they can receive the best therapy against cancer.

## Background

Cardio-oncology is a relatively new area of activity in Brazil and in the world. The importance of cardio-oncology has been growing over recent years due to the increase in the number of cancer patients and survivors with cardiovascular disease (CVD). Heart disease and cancer are the leading causes of mortality worldwide and have several common risk factors [1, 2].

In recent decades, significant advances have changed the history of cancer patients, and we can observe an increasing number of survivors. By 2026, data from the USA suggest that 20.3 million people will survive cancer [2, 3]. The injuries caused to the cardiovascular system fall within a spectrum and might affect all cardiovascular structures, with manifestations ranging from asymptomatic disease to cardiovascular death [4]. Therefore, a structured programme aimed at caring for these patients has the potential to evaluate cancer patients with safety and quality during different phases of therapy.

This article describes the experience of a pioneer programme in Brazil. The Instituto do Cancer do Estado de Sao Paulo (ICESP) belongs to the University of São Paulo and was founded in 2008. Today, it is the largest hospital in Latin America for cancer treatment. Due to the high cardiovascular risk profile of cancer patients and the increased incidence of cardiovascular disease, in May 2009, the ICESP Cardio-Oncology Programme was created. Annually, this hospital registers approximately 8000 new cases of cancer patients, delivers 46,000 chemotherapies and performs 7800 oncologic surgeries. In 2017, we created a cardio-oncology fellowship, aiming to specialize cardiologists in the cardiovascular care of cancer patients.

In this article, we describe the 10 years of the Cardio-Oncology Programme, highlighting its performance in health care as well as in education and research. In addition, we show unique opportunities and challenges associated with the development of a cardio-oncology programme in a low-income country.

## Methods

A retrospective study was carried out with the information obtained from Health Information Management, a database of the Instituto do Cancer do Estado de Sao Paulo (ICESP). ICESP is a public university hospital specialized in the care of cancer patients and has a wide structure that allows cardiovascular management of these patients, such as prevention, diagnosis and therapy for complications. ICESP is coordinated by a board of directors linked to the University of São Paulo Medical School (FMUSP). It is located within the “Hospital das Clinicas” and is physically linked to the heart institute (Instituto do Coração/InCor).

We conducted a survey of all outpatient care provided by the cardiology department in the hospital from April 2009 to February 2019. This article has been approved by the local ethics committee. Since May 2013, appointments have been registered in a structured template that allows patient information to be recorded and information to be exchanged among specialists. Information regarding comorbidities, oncologic diagnosis, cancer therapy, and imaging data of the patients was collected from this database.

Clinical and surgical specialists refer patients to the Cardio-Oncology Programme. Patients are initially screened by the assistants through a checklist of indications (Table 1). Both outpatient and inpatient service is performed by one of the members of cardio-oncology and by the cardiology resident, supervised by cardio-oncology physician assistants. Once a week, patients are evaluated in a general visit with the cardio-oncology professor, and two clinical sessions are performed a week with case discussions and a journal club.

**Table 1:** Follow-up criteria in the Cardio-Oncology Service

**Required Criteria**	
Confirmed Cancer Diagnosis	
**Additional Criteria**	
Presence of uncontrolled or multiples cardiovascular risk factors: hypertension, diabetes, smoker, obesity, elderly	
Heart Disease: Arrhythmia, coronary artery disease, heart failure, infiltrative disease, venous and arterial thromboembolism, arterial hypertension,	
Chemotherapy com cardiotoxic potential: anthracycline, monoclonal antibodies (trastuzumab and bevacizumab), anti-VEGF inhibitors, multi-targeted tyrosine kinase inhibitors, small molecule tyrosine kinase inhibitors, proteasome inhibitors, immunotherapy, gonadotropin agonist.	
Perioperative evaluation: major surgery (thoracic, abdominal, vascular, genitourinary and gynecological) or presence of cardiovascular symptoms (dyspnea, chest pain, syncope) or structural heart disease (valvular disease, dilated and/or restrictive/infiltrative cardiomyopathy, coronary artery disease, pulmonary thromboembolism or heart failure).	

The medical assistant of the Cardio-Oncology Service assesses the request for assistance from other medical specialties. Patients with cardiovascular risk factors or cardiovascular disease are referred to a general medical care outpatient. When cardiotoxicity is suspected or confirmed, the patients are referred to the cardiotoxicity medical care outpatient. If cardiotoxicity is not confirmed and the patient has an indication for follow-up with Cardio-Oncology, the patient is referred to the general clinic. Cases with indication for surgical treatment are evaluated in the Perioperative outpatient clinic

**Fig. 1:**
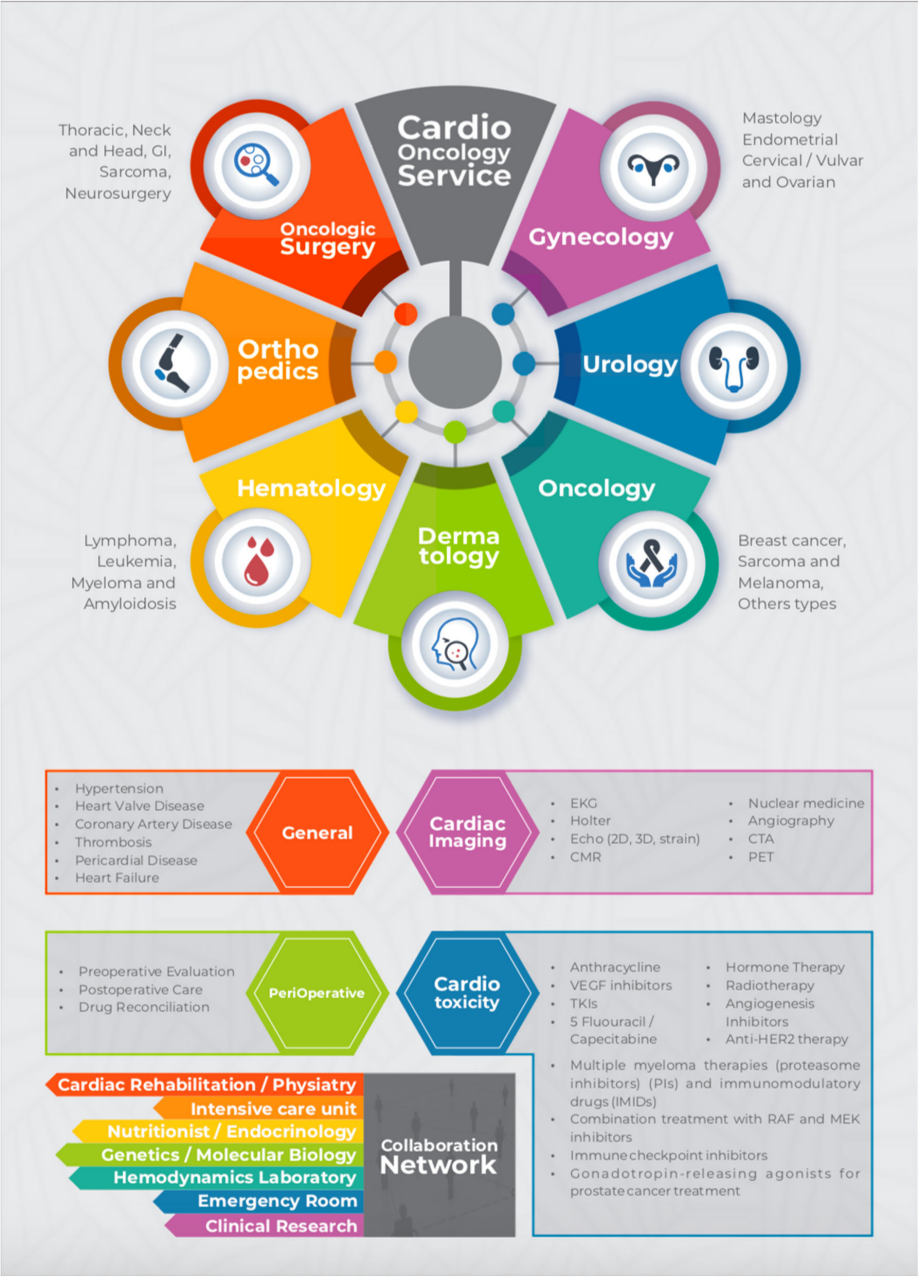
Patients followed by clinical and surgical oncology medical teams are referred to the Cardio-Oncology Programme. To organize the programme, patients are referred to outpatient clinics initially separated by the reason for requesting referral: general, cardiotoxicity and perioperative. The cardiac imaging sector is interconnected with the outpatient programme. According to their clinical indication, patients can be evaluated by the collaborative network that follows in partnership with the clinical team. CMR, cardiac magnetic resonance; CTA, computed tomography angiography; EKG, electrocardiogram; GI, gastrointestinal; HER2, human epidermal growth factor receptor; IMIDs, immunomodulatory drugs; PET, positron emission tomography; PI, proteasome inhibitors; TKI, tyrosine kinase inhibitors; VEGF, vascular endothelial growth factor

The general outpatient clinic aims to treat cancer patients with chronic heart disease or cardiovascular risk factors that develop and require cardiac follow-up. The perioperative outpatient clinic is designed to assist the patient in all stages of surgical planning. The cardiotoxicity outpatient clinic focuses on treating complications from chemotherapy or radiotherapy treatment to resolve these complications. The central illustration summarizes the cardiology care and multidisciplinary flow, which is the basis of the programme (Fig. 1).

### Statistical analysis

Categorical variables were compared using Pearson Chi-square tests, Fisher exact tests, or likelihood ratio tests. Continuous variables were compared using analysis of variance and Tukey’s test (normal distribution) or Kruskal-Wallis and Dunn’s tests. The results are expressed as the means with standard deviations or as the medians with interquartile ranges. Linear correlations were tested using the Spearman rank method. All analyses were performed using SPSS version 17 (SPSS, Inc., Chicago, Illinois, USA). A *p* value < 0.05 was considered statistically significant.

## Results

**Fig. 2:**
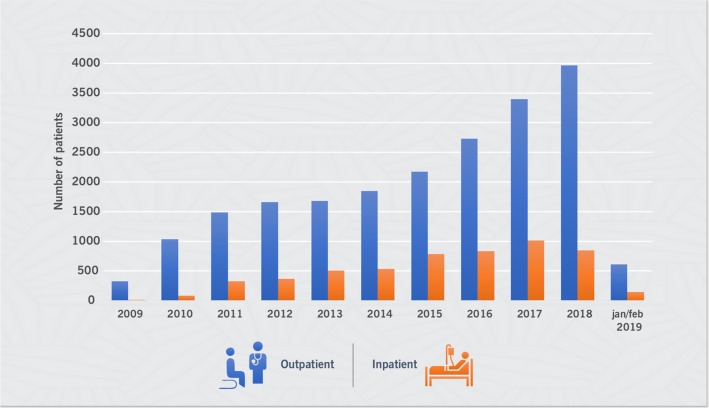
Number of inpatients and outpatients seen over the years

**Fig. 3:**
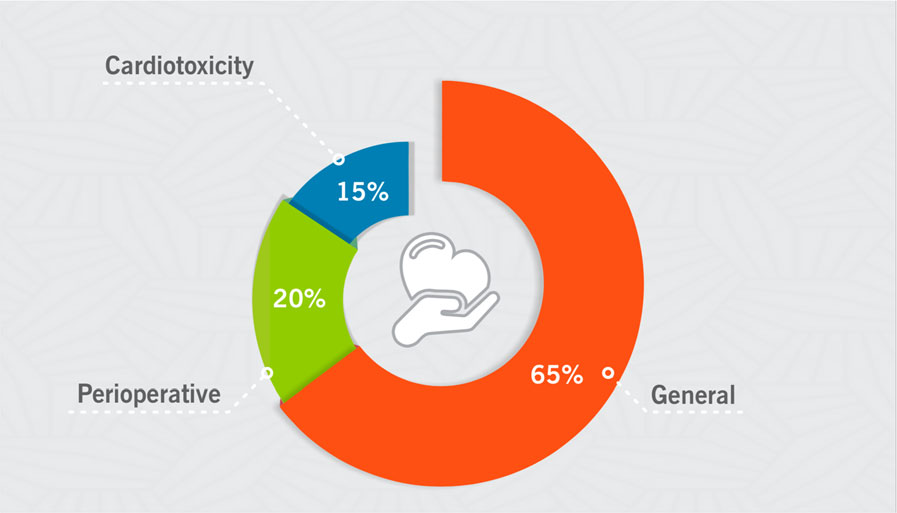
Distribution of outpatients by type of care

During the 10 years of cardio-oncology practice, a total of 20,991 outpatient medical care and 5444 inpatient medical care sessions were performed (Fig. 2). There has been exponential growth in the number of patients provided care over the years. There were 14,990 outpatient visits (corresponding to the care of 4662 patients) as of May 2013, distributed into outpatient clinics as follows: 9706 (65%) general, 2943 (20%) perioperative and 608 (15%) cardiotoxicity (Fig. 3).

**Fig. 4:**
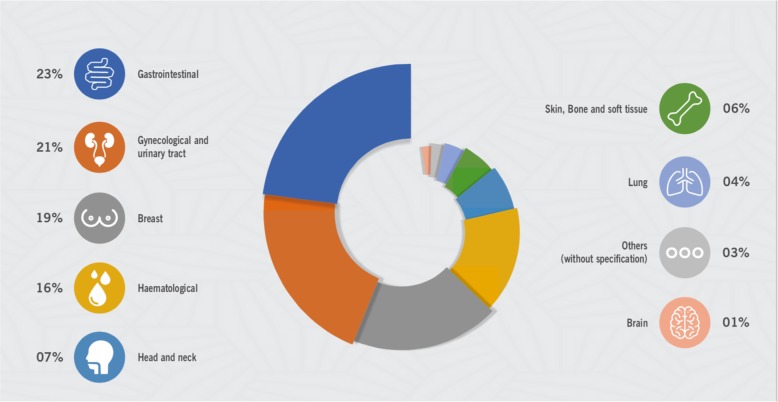
Distribution of outpatients by type of cancer

Of the total number of patients, 4525 had their clinical characteristics recorded in the template. Table 2 summarizes the main characteristics of the patients. There is a high prevalence of cardiovascular risk factors in this population, especially hypertension, detected in 2735 (60.4%) patients; dyslipidaemia, detected in 1233 (22.4%) patients; and smoking, detected in 1840 (40.7%) patients. The predominant types of cancer in this population were gastrointestinal in 1039 patients (23%), gynaecological/urological in 947 patients (21%) and breast cancer in 856 patients (19%)(Fig. 4).

**Table 2:** Baseline characteristics of patients

Characteristics of patients	Total
	*n* = 4525
Male, n (%)	1939 (43)
Age (years), median and IQR	66 (58–74)
Diabetes, n (%)	1081 (23.9)
Hypertension, n (%)	2735 (60.4)
Dyslipidemia, n (%)	1233 (22.4)
Current smoking, n (%)	369 (8.2)
Previous smoker, n (%)	1471 (32.5)
Obesity, n (%)	441 (9.7)
Cerebrovascular Disease, n (%)	276 (6.0)
Hyperuricemia, n (%)	70 (1.6)
Carotid Disease, n (%)	37 (0.8)
Peripheral arterial disease, n (%)	72 (1.6)
Tachyarrhythmias, n (%)	111 (2.4)
Atrial fibrillation / flutter, n (%)	385 (8.5)
Bradyarrhythmias, n (%)	165 (3.6)
Amyloidosis, n (%)	18 (0.4)
Coronary artery disease, n (%)	616 (13.6)
Pulmonary thromboembolism, n (%)	106 (2.4)
Aortic valve disease, n (%)	145 (3.2)
Aortic aneurysm, n (%)	45 (1.0)
Mitral Valve Disease, n (%)	112 (2.4)
Ischemic Cardiomyopathy, n (%)	366 (8.1)
Hypertensive Cardiomyopathy, n (%)	54 (1.2)
Hypertrophic Cardiomyopathy, n (%)	14 (0.3)
Chagas Cardiomyopathy, n (%)	73 (1.6)
Dilated / idiopathic Cardiomyopathy, n (%)	273 (6.0)
Primary cardiac tumors, n (%)	10 (0.2)
Echocardiographic data, median and IQR:	
AO (mm)	33 (30–37)
LA (mm)	38 (34–43)
Septo (mm)	10 (9–11)
PW (mm)	9 (9–10)
LVEDD (mm)	48 (43–52)
LVESD (mm)	32 (29–38)
PSAP (mm)	33 (28–41)
LVEF (%)	62 (52–66)

*AO* Aorta, *LA* Left atrium, *PW* Posterior wall, *LVEDD* Left ventricular end-diastolic diameter, *LVESD* Left ventricular end systolic diameter, *LVEF* Left ventricular ejection fraction, *ITQ* Interquartile range, *PSAP* Pulmonary artery systolic pressure, *IQR* Interquartile range

Clinical characteristics were separated by type of care, as shown in Table 3. When analysing the initial patient profile of the general cardiology, perioperative follow-up and treatment cardiotoxicity outpatient clinics, differences were observed with respect to age (59 [48–66], 66 [58–74] and 69 [62–76], *p* < 0.001), diabetes (67 [15%], 635 [22.6%] and 379 [29.8%]; *p* < 0.001), hypertension (196 [43.8%], 1649 [58.7%] and 890 [70.1%], *p* < 0.001) and dyslipidaemia (87 [19.7%), 735 [26.2%] and 459 [36.2%], *p* < 0.001). These results show that the cardiotoxicity group has a lower prevalence of cardiovascular risk factors than the other groups. The type of cardiotoxicity most common in our cohort was ventricular dysfunction, present in 253 patients (57.1%). The median left ventricular ejection fraction at the time of diagnosis was 52 (40–58)%. We observed a lower LVEF in the cardiotoxicity group than in the general and perioperative groups (55 [41–63]%, 62 [55–66]%, 62 [55–66]%, *p* < 0.001).

**Table 3:** Comparison of clinical characteristics according to the type of outpatient care

Variable	Cardiotoxicity	General	Perioperative	p
	*n* = 448	*n* = 2807	*n* = 1270	
Male, n (%)	105 (23.4%)	1242 (44.2%)	592 (46.6%)	< 0.001ª
Age (years), median and IQR	59 (48–66)	66 (58–74)	69 (62–76)	< 0.001^b^
Cancer Type				< 0.001ª
Gastrointestinal	39 (8.7%)	679 (24.2%)	321 (25.3%)	
Breast Cancer	237 (52.9%)	510 (18.2%)	109 (8.6%)	
Hematological	118 (26.3%)	574 (20.4%)	21 (1.7%)	
Head and neck	5 (1.1%)	183 (6.5%)	143 (11.3%)	
Skin, bone and soft tissue	12 (2.7%)	110 (3.9%)	156 (12.3%)	
Brain	0 (0%)	15 (0.5%)	6 (0.5%)	
Lung	6 (1.3%)	148 (5.3%)	44 (3.5%)	
Gynecological and urinary tract	20 (4.5%)	505 (18%)	422 (33.2%)	
Other / without specification	11 (2.5%)	83 (3%)	48 (3.8%)	
Diabetes	67 (15%)	635 (22.6%)	379 (29.8%)	< 0.001ª
Hypertension	196 (43.8%)	1649 (58.7%)	890 (70.1%)	< 0.001ª
Dyslipidemia	87 (19.7%)	687 (25.7%)	459 (36.2%)	< 0.001ª
Current smoking	29 (6.5%)	214 (8%)	126 (9.9%)	0.086ª
Previous smoker	107 (24.2%)	907 (33.9%)	457 (36%)	< 0.001ª
Obesity	49 (11%)	262 (9.8%)	130 (10.3%)	0.343ª
Hyperuricemia	9 (2%)	33 (1.2%)	28 (2.2%)	0.091ª
Carotid Disease	2 (0.5%)	20 (0.7%)	15 (1.2%)	0.414ª
Peripheral arterial disease	3 (0.7%)	30 (1.1%)	39 (3.1%)	< 0.001ª
Cerebrovascular Disease	11 (2.5%)	171 (6.4%)	94 (7.4%)	0.003ª
Atrial fibrillation / flutter	15 (3.4%)	270 (10.1%)	100 (7.9%)	< 0.001ª
Coronary artery disease	22 (4.9%)	358 (13.5%)	236 (18.6%)	< 0.001ª
LVEDD (mm), median and IQR	49 (45–53)	47 (43–51)	49 (44–53)	< 0.001^b^
LVESD (mm), median and IQR	35 (31–43)	32 (29–38)	32 (29–38)	< 0.001^b^
LVEF (%), median and IQR	55 (41–63)	62 (55–66)	62 (55–66)	< 0.001^b^

a: Chi-squared test, b: Kruskal-Wallis test

*LVEDD* Left ventricular end-diastolic diameter, *LVESD* Left ventricular end systolic diameter, *LVEF* Left ventricular ejection fraction, *ITQR* Interquartile range

Similar overall mortality rates of 47.5, 45.7 and 44.9% (*p* = 0.650) were observed in the groups. The mean follow-up period was 7.7 ± 2.7 years.

## Discussion

From the results of a follow-up period of almost 8 years, we report that cancer patients referred to a referral cancer institute in Brazil present a high prevalence of cardiovascular risk factors and that compared to other patients, patients with treatment-induced cardiotoxicity present the lowest left ventricle ejection fraction and a lower number of cardiovascular risk factors. In addition, the mortality rate of these patients is high and not associated with the cardiovascular disease setting.

### Cardiotoxicity

The profile of a patient with cardiotoxicity reflects a younger population with fewer risk factors and predominant breast cancer. This population analysis suggests that the cardiac complications found in these patients resulted from cancer therapy. Patients in this group had a lower left ventricular ejection fraction, despite presenting fewer comorbidities.

Care in this outpatient clinic is focused on all forms of cardiotoxicity (ventricular dysfunction, myocardial ischaemia, hypertension, arrhythmias); however, the vast majority is related to anthracycline and trastuzumab cardiotoxicity, which lead to ventricle dysfunction. The largest Brazilian study conducted in our institution estimated the incidence of anthracycline-related cardiotoxicity in breast cancer patients to be approximately 14% [5].

The concept of cardiotoxicity has changed over the years. Initially, only the ejection fraction drop was valued as a criterion. The I Brazilian Cardio-Oncology Guideline of the Brazilian Society of Cardiology [6], coordinated by the ICESP and InCor teams, redefined cardiotoxicity as follows: 1) cardiomyopathy with reduced left ventricular ejection fraction (LVEF), 2) heart failure (HF) symptoms, 3) HF-associated signs such as S3, tachycardia or both; 4) reduction in LVEF compared to a baseline of at least 5% to less than 55% with concomitant signs or symptoms of HF, or reduction in LVEF in the range of at least 10% to less than 55% without signs or concomitant symptoms. In recent years, the programme has incorporated the currently accepted definition of cardiotoxicity as a drop-in ejection fraction of at least 10% absolute points to values below 50% [7]. The standard treatment involves angiotensin-converting-enzyme inhibitors (ACEIs) and/or angiotensin II receptor blockers (ARBs) associated with beta blockers (BBs) and spironolactone. The maintenance of chemotherapy can be considered in this scenario but with cardiac imaging monitoring and biomarkers. There is not an exact definition as to when chemotherapy should be halted – this decision should preferably be made alongside the assistant team (oncologist or haematologist) [7].

One of the key tools for deciding how to select the cardioprotection strategy for a certain patient is based on a complete clinical history and complete physical examination, the identification of cardiovascular (CV) risk factors, biomarker analysis, and an evaluation of the potential risk of cardiotoxicity [8]. It is also important to discuss with the cancer team the type of chemotherapy to be administered, its dose and its potential cardiovascular toxicities. Following this initial approach, optimization of CV risk factors and previous CV conditions should be achieved following its specific therapeutic goals [7, 8].

### General cardiology

The incidence of cardiovascular risk factors is high in this population. The median age was 66 (58–74) years, which shows that most of the patients attended are elderly. Age is an isolated risk factor and contributes to the development of CVD and cancer. The risk for CVD doubles with each decade of life, and it is estimated that 70% of new cancer cases occur in individuals over 55 years of age [9, 10].

The prevalence of hypertension in our population is high, with approximately 60% of patients exhibiting hypertension. The treatment for hypertension in oncological patients must follow the recommendations from current guidelines [11, 12]. The main objective of antihypertensive treatment for oncological patients aside from reducing cardiovascular risk is to enable them to receive antineoplastic treatment at maximum doses and ultimately derive benefits from neoplasm control.

Cardiac arrhythmia is another frequent complication with oncological patients, since it can be a side effect to different drugs [13, 14]. Aside from antineoplastic therapy, it is necessary to be cautious about a series of factors present in oncological patients that can contribute to this exponential incidence, such as electrolyte disorders, hypothyroidism, advanced age, and concomitant use of drugs that prolong QTc. These conditions must be treated and corrected for all patients who will initiate chemotherapeutic treatment [7].

The main drugs related to prolonged QT are arsenic trioxide and tyrosine kinase inhibitors. When these drugs are administered, a basal electrocardiogram must be repeated periodically during chemotherapy. The discontinuation of chemotherapy is recommended when QTc prolongation is > 500 ms or there is an increase of more than 60 ms in basal QTc [7, 15, 16].

Supraventricular arrhythmia is also relatively frequent in oncological patients, where atrial fibrillation (AF) is most commonly noticed [14]. In our cohort, the prevalence of atrial fibrillation was 8.74%. Elevated incidence of AF is acute in scenarios of post-procedures, and the treatment in these cases is indicated [17].

The incidence of thromboembolism (VTE) phenomena in cancer patients is high, and VTE is the second leading cause of mortality in these patients [18]. For patients with documented thromboembolic events, full dose anticoagulation could be conducted for those with platelet counts > 50,000/mm^3^, with no associated coagulation disorders [19]. Coagulation in patients with active neoplasms and/or ongoing chemotherapy treatment can be performed, preferably with low molecular weight heparin, avoiding the use of warfarin in this context [19].

Recent published studies have tested the efficacy and safety of direct-acting oral anticoagulants (DOAC) for VTE treatment and prophylaxis in cancer patients. The results show that DOACs are viable alternatives, as long as they are used with caution in patients who are taking drugs with high drug interactions or have gastrointestinal tumours or severe renal dysfunction [20–22]. In ICESP, the use of a DOAC (rivaroxaban) was regularized in 2018 and is regularly provided to patients with indication of its use.

Another important complication in cancer patients is coronary artery disease (CAD). Approximately 15% of all patients had documented CAD on their first visit to ICESP. The definition of CAD treatment in oncology patients must be performed quickly to avoid intermission or interruption of oncological disease treatment. The decision between clinical or invasive treatments (percutaneous or surgical) must follow current protocol recommendations and be decided upon preferably only after clinical consultation with the entire team (cardiologist, haematologist, oncologist and surgeons).

The high mortality rate observed in this patient cohort reflects the complexity and severity of these patients in long-term follow-up. CVD and cancer are the leading causes of death worldwide. The corresponding estimates for total cancer deaths in 2007 are 7.6 million (approximately 20,000 cancer deaths a day). In 2018, the corresponding estimated cancer deaths were 9.5 million higher (approximately 26,000 cancer deaths a day). By 2040, the projected 16.3 million cancer deaths will be simply due to the growth and ageing of the population [10].

### Perioperative evaluation and follow-up

In ICESP, there is a high volume of cancer surgery. In our cohort, we observed a high prevalence of cardiovascular risk factors in this group of patients. The most frequent tumour types evaluated in this patient group were gynaecological/urological and gastrointestinal tumours. Evidence suggests that patients undergoing surgery for a cancer indication have a substantially increased risk of fatal pulmonary embolism compared to that in patients undergoing surgery for benign disease [23]. The risk of bleeding in cancer patients is also elevated and multifactorial and is a complication of several surgical procedures [24].

The risk of perioperative complications depends on the clinical condition of the patients before surgery, prevalence of comorbidities, urgency, magnitude, type and duration of the surgical procedure. Each surgery provokes a different level of surgical stress [25]. The most difficult aspect in the perioperative evaluation of these patients would be time. The scheduled oncological surgery does not fit an urgent situation, but it also cannot be considered totally elective. If surgery is delayed, the tumour may grow, and the cancer staging may change. Thus, oncological surgery can be classified as “time sensitive”, with the best results if performed between 1 and 6 weeks after indication [26].

The evaluation aims to minimize the risks through an individualized evaluation that integrates the risk factors with the surgical stress of the planned procedure. In this way, the cardiovascular risk can be estimated with the opportunity to administer medications, coronary interventions when necessary, and planning of the most suitable surgical and anaesthetic techniques. Among the main cardiovascular complications observed in patients under cancer treatment, the most common are HF, myocardial ischaemia, hypertension, arrhythmia and thromboembolism [26].

### Structure of the cardio-oncology programme

#### Cardiac imaging

At ICESP, drugs with cardiotoxic potential are followed according to standard institutional protocols. Follow-up protocols exist for anthracyclines, anti-HER2, vascular endothelial growth factor (VEFG) inhibitors (angiogenesis inhibitors, tyrosine kinase inhibitors and multi-target), BCr-Abl inhibitors, proteasome inhibitors and immunomodulatory drugs, immunotherapy and gonadotrophin agonists (Fig. 1).

Routine exams performed on the patients comprise 12-lead electrocardiogram (EKG) and 2D-echocardiography with myocardial strain. The electrocardiogram should be performed in all patients who will be submitted to drugs with cardiotoxic potential. Patients receiving 5-fluorouracil and capecitabine who are at risk of developing vasospasm and arrhythmias during use should have a basal and repeated EKG whenever cardiovascular symptoms are present or at the end of the treatment. In patients using drugs with a moderate-to-high risk of developing QTc interval prolongation, such as bosutinib, arsenic trioxide, sunitinib, sorafenib and dasatinib, a baseline electrocardiogram is recommended every 3–4 weeks and at the end of the treatment. A 24-h Holter can be used to monitor these patients when there is a strong suspicion of arrhythmia.

Transthoracic echocardiography is performed on all patients who receive drugs with cardiotoxicity potential, such as anthracyclines and trastuzumab. Patients who are treated with anthracyclines undergo an echocardiogram before treatment, at the end of treatment and within 1 year. Patients with high-dose schedules (> 300 mg/m^2^) are usually re-evaluated mid-treatment. Patients who are at high risk of developing cardiotoxicity, with limited or early initial ejection fraction or structural heart disease, are closely monitored with echocardiography. For patients treated with trastuzumab, we perform echocardiography before the start of treatment, at 24 weeks, and at the end of treatment.

Cardiac magnetic resonance imaging plays a special role in patients with cancer, mainly to define the aetiology of ventricular dysfunction in these patients [27]. Takotsubo syndrome, myocarditis, infiltrative disorders and ischaemic disease are common in cancer patients and should be differentiated from cardiotoxicity [27, 28]. Patients with suspected immunotherapy-induced myocarditis should undergo cardiac magnetic resonance imaging for diagnostic investigation.

Coronary artery computed tomography and myocardial scintigraphy are diagnostic tools frequently used with cancer patients to stratify coronary artery disease. Patients with cardiovascular risk factors and/or cancer therapy related to atherosclerosis/coronary thrombosis (such as cisplatin, some TKI, radiotherapy) are usually investigated with these tests. Invasive angiography is especially important in symptomatic patients and in acute coronary syndrome. When indicated, cardiac biopsy, transcatheter aortic valve replacement, and balloon valvuloplasty were performed in the haemodynamic laboratory at InCor.

#### Collaboration network

The Cardio-Oncology Programme works in partnership with a large collaboration network trying to provide comprehensive care for patients. All patients treated at the cardio-oncology outpatient clinic were accompanied by a nursing evaluation. Nursing care aims to assist in demands related to therapeutic adherence and personal care. Patients who present acute cardiac complications during their follow-up at ICESP are treated at the emergency room or intensive care unit, and the cardio-oncologists visit these patients daily and follow-up in conjunction. The cardio-oncologist assists in the diagnosis and therapeutic decision of these patients.

When indicated, patients undergo nutritional evaluation and have an endocrinologist to assist with metabolic disorders. Patients with ventricular dysfunction and coronary artery disease are referred for physical therapy and cardiac rehabilitation. Supervised training and guidance for home training are provided. There is a clinical research unit at ICESP that conducts clinical and experimental research (genetic research and molecular biology). In addition, palliative care is structured in a specialized programme.

#### Cardio-oncology fellowship programme

The Cardio-Oncology Fellowship Programme was established in 2017 as a one-year programme offered to physicians with previous cardiology backgrounds. It aims to train cardiologists (6 per year) so they can properly detect and treat cardiovascular complications in cancer patients. The theoretical basis of the programme consists of two weekly meetings. The first meeting aims to offer the members a better understanding of cancer treatments and the clinical course so they can properly manage concurrent cardiovascular complications. The second meeting consists of clinical case discussions followed by literature reviews of major cardio-oncology topics presented by the members of the programme.

The practical programme covers four main areas.

1. Inpatient clinic: members rotate in the inpatient clinic for 4 months, managing cardiac issues of cancer patients.
2. Outpatient clinic: fellows rotate for 4 months.
3. Clinical oncology and haematology: members rotate in both oncology and haematology outpatient clinics for 1 month.
4. Optional internship: an optional month is provided for the members, so they are able to visit other cardio-oncology clinics in Brazil or even internationally.

The members progress through these four main areas following a monthly prespecified rotation schedule. The cardio-oncology department also receives cardiology residents from other centres, such as Hospital Sírio Libanes and InCor, for monthly rotation periods.

## Limitations

This is a retrospective analysis from a unique cancer hospital, but the evaluation of a large number of patients coming from a reference cancer institute has merit and the potential for generalizability. We did not analyse the total sample because we could not retrieve data before 2013. However, these massive numbers add knowledge to the field, with the aim of showing health professionals how to launch a cardio-oncology programme.

## Conclusions

The Cardio-Oncology Programme has grown significantly in the last decade as a result of higher survival rates and a greater number of referred patients. These data suggest a growing awareness about the importance of managing cardiovascular disease in these patients, with respect to preventing such disease and performing early diagnosis and treatment.

A specialized programme allows new professionals to be trained, knowledge to be shared, and innovative research to be developed and results in a productive interaction between cardiologists and oncologists to provide safe and efficient therapy for cancer patients.

## Data Availability

All data generated or analysed during this study are included in this published article. If you have questions or additional information, the datasets used and/or analysed during the current study are available from the corresponding author upon reasonable request.

## Abbreviations

ACEI: Angiotensin-converting-enzyme inhibitors
AF: Atrial fibrillation
ASA: Acetylsalicylic acid
ARBs: Angiotensin II receptor blockers
BB: Beta blockers.
CAD: Coronary artery disease
CV: Cardiovascular
CVD: Cardiovascular disease
DOAC: Direct-acting oral anticoagulants
EKG: Electrocardiogram
FMUSP: University of São Paulo Medical School
HF: Heart failure
InCor: Instituto Coracao/heart institute
ICESP: Instituto do Cancer do Estado de São Paulo
LV: Left ventricle
LVEF: Left ventricular ejection fraction
S3: Third heart sound
TKI: Tyrosine kinase inhibitors
VEGF: Vascular endothelial growth factor
VTE: Thromboembolism
